# Optical engineering of PbS colloidal quantum dot solar cells via Fabry–Perot resonance and distributed Bragg reflectors

**DOI:** 10.1186/s40580-023-00379-1

**Published:** 2023-07-04

**Authors:** Sumin Bae, Matthew Duff, Jun Young Hong, Jung-Kun Lee

**Affiliations:** Department of Mechanical Engineering and Materials Science, University of Pittsburgh, Pittsburgh, PA 15261 USA

**Keywords:** Colloidal quantum dots, Solar cells, Light trapping, Wavelength selectivity, Fabry–Perot resonance, Distributed Bragg reflectors

## Abstract

A tradeoff between light absorption and charge transport is a well-known issue in PbS colloidal quantum dot (CQD) solar cells because the carrier diffusion length in PbS CQD films is comparable to the thickness of CQD film. We reduce the tradeoff between light absorption and charge transport by combining a Fabry–Perot (FP) resonator and a distributed Bragg reflector (DBR). A FP resonance is formed between the DBR and a dielectric-metal-dielectric film as a top transparent electrode. A SiO_2_-TiO_2_ multilayer is used to form a DBR. The FP resonance enhances light absorption near the resonant wavelength of the DBR without changing the CQD film thickness. The light absorption near the FP resonance wavelength is further boosted by coupling the FP resonance with the high reflectivity of the Ag-coated DBR. When the FP resonance and DBR are combined, the power conversion efficiency (PCE) of PbS CQD solar cells increases by 54%. Moreover, the DBR assisted FP resonance enables a very thin PbS layer to absorb near infrared light four times more. The overall PCE of the thin PbS CQD solar cell increases by 24% without sacrificing the average visible transmittance (AVT). Our results show how to overcome the inherence problem of the CQD and develop a semi-transparent solar cell where the wavelength-selective absorption and the transparency for visible light are important.

**Supplementary Information:**

The online version contains supplementary material available at 10.1186/s40580-023-00379-1.

## Introduction

Colloidal quantum dots (CQDs) have been extensively explored for the application of a next-generation solar cell. CQDs enable a low-cost solution process and have an advantage in utilizing a wide spectral range of solar energy by adjusting their bandgap [1, 2]. Lead chalcogenide (PbS and PbSe) CQDs have received much attention due to their excellent optical properties and handy synthetic methods for easy tuning of particle size [3]. In the past few years, CQD solar cell technologies have grown rapidly and the best power conversion efficiency (PCE) of the PbS CQD solar cell is over 13% [4].

One fundamental question of PbS CQD solar cells is how to improve their charge transport properties that are mainly influenced by the charge hopping between CQDs. The charge transport of CQDs are controlled by exciton dissociation, hopping between CQDs, trapping, and recombination steps [5]. As the thickness of the CQD layer increases, more light is absorbed, and more charge carriers are produced. However, an increase in the CQD layer thickness increases the probability of the charge recombination at the trapping sites such as the interface between CQDs. This energy loss in a form of heat decreases the power conversion efficiency (PCE) of the solar cells. Thus, to boost the light absorption without suppressing the carrier transport is important in designing the device structure of the CQD solar cells. Research efforts to address this challenge are classified into two groups. One is to enhance the carrier transport by reducing the trapping at the interface, and the other is to increase the light absorption coefficient of CQDs. The former one is usually achieved by replacing long hydrocarbon chains on the surface of the CQDs with short functional ligands. By reducing interdot distances between quantum dots using shorter ligands, carrier mobility and lifetime product can be increased [6, 7]. In addition, the ligand exchange passivates surface trap sites such as dangling bonds [8] and controls energy band alignment at the interfaces [9]. However, these ligand exchange methods are typically complex and require precise optimization to achieve desired performance.

The device architecture can be engineered to absorb more light while maintaining CQD film thickness. Fabry–Perot (FP) resonator is an effective optical structure to overcome the limited light absorption of thin active layers that have a problem of carrier trapping. A FP resonator is an optical cavity where light oscillates between two highly reflecting mirrors. This oscillation assists photoactive materials in absorbing more light at the FP resonant wavelength [10]. It is worth noting that the FP effect is more significant with thin active layer devices [11]. Therefore, the light trapping using the FP effect is very promising for solar cells that need to have a very thin active layer such as semi-transparent solar cells.

PbS CQDs can be controlled to allow some of the visible light to pass through, while absorbing light from ultraviolet (UV) to near infrared (NIR) region due to the quantum confinement effect. Although this characteristic absorption spectrum makes the PbS CQDs advantageous to be utilized as a light absorbing material of semi-transparent solar cells, only a few studies have been reported [12–16]. In all the previous studies, visible transparency was secured by reducing the thickness of PbS active layer. However, the resulting red to NIR absorption was significantly decreased, resulting in a relatively low photo-current generation. The FP resonance effect can address this trade-off between the thickness of active layer and optical transparency. Moreover, utilizing a distributed Bragg reflector (DBR) can further boost the light absorption without sacrificing visible transparency by selectively enhancing light absorption in a non-visible wavelength range.

**Fig. 1:**
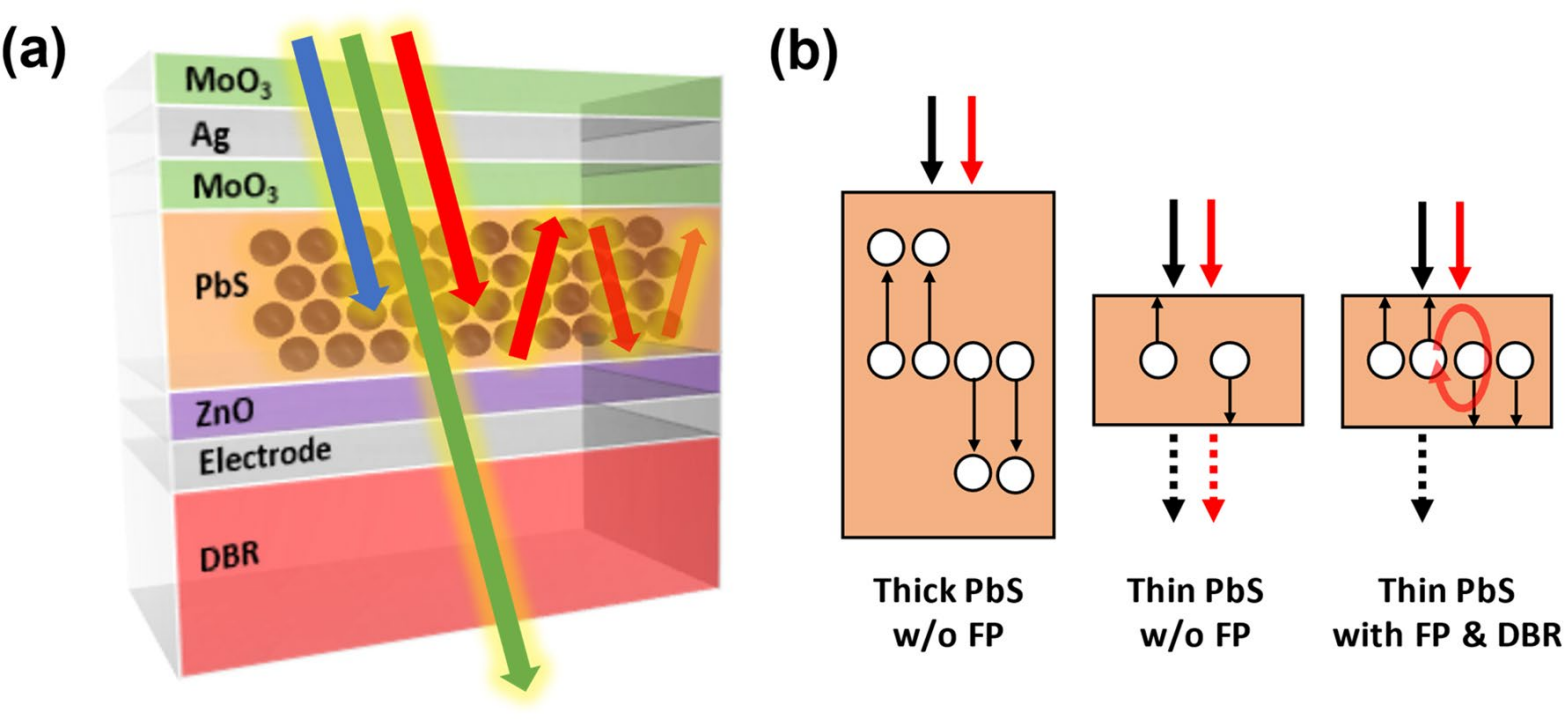
Schematic of **a** the distributed Bragg reflector (DBR) assisted Fabry–Perot (FP) resonance integrated solar cell, and **b** charge transport illustration in thick PbS active layer without FP, thin PbS without FP, and thin PbS with DBR assisted FP

Here, we report a rational design to enhance the light absorption of PbS CQD solar cells. Though the PbS layer is thin, the combination of FP resonance and DBRs promotes the light absorption and increases an incident photon to current efficiency (IPCE) of the solar cells. The device design is shown in Fig. 1a. In this structure, light is illuminated through the transparent dielectric-metal-dielectric (DMD) electrode. Ag-coated DBR consisting of alternating TiO_2_ and SiO_2_ films is below the PbS layer. The Ag-coated DBR offers a very high reflectivity in the selected wavelength. In addition, the FP resonance is generated between the Ag layer of the DMD film and a bottom Ag layer. The coupling of the FP resonance with the DBR significantly increases the light absorption of the CQD layer without sacrificing the charge transport as shown in Fig. 1b. This leads to the improvement of the PCE by 54% and IPCE by 215% at the resonant wavelength in comparison to a reference device. In addition, we demonstrate that the PbS CQD solar cell with FP and DBR effects can be used as a semi-transparent solar cell. IPCE of PbS thin film solar cells with FB and DBR effects increases from 8.3 to 44.9% at the resonant wavelength in the NIR region where the weak absorption valley exists. Consequently, the overall PCE of the thin film solar cell increases by 24% while AVT remains almost the same. This shows the potential of developing a new class of semi-transparent solar cells using CQDs.

## Material and methods

Lead oxide (PbO, 99.99%), 1-octadecene (ODE, 99%), and oleic acid (OA, 99%) were purchased from Alfa Aesar. Bis(trimethylsilyl)sulfide (TMS, 97%) was purchased from TCI America. Zinc acetate dihydrate (98%), potassium hydroxide (85%), lead iodide (PbI_2_, 99%), lead bromide (PbBr_2_, 98%), ammonium acetate (NH_4_Ac, 98%), acetonitrile (99.8%), and 1,2-ethanedithiol (EDT, 98%) were purchased from Sigma-Aldrich.

### Synthesis of PbS CQDs

The synthesis of oleic-acid-capped PbS CQD was performed using a previously published method [17]. ODE was degassed under vacuum at 85 °C for 12 h. 450 mg of PbO, 18 mL of ODE, and 1.5 mL of OA were mixed in a three-neck flask under vacuum at 70 °C for 2 h. Under air-free Schlenk line conditions, the atmosphere of the flask was switched to nitrogen. In a nitrogen ambiance, the temperature of the mixture was raised from 70 to 120 °C for an hour. After the mixture became clear at 120 °C, a syringe containing 10 mL of ODE and 211 µL of TMS was prepared while temperature of the solution in the flask was set to the injection temperature (80–150 °C). To initiate the PbS nucleation, the solution of ODE and TMS was swiftly injected into the flask. Temperature of the solution in the flask was monitored with an in-situ thermocouple. After the injection, the flask was immediately removed from an oil bath, and the sudden drop in temperature followed by the crystal growth of the PbS CQDs. The solution cooled naturally to room temperature under nitrogen flow for 30 min. As-synthesized PbS CQDs were separated by centrifugation with acetone and dispersed in hexane. Prior to the device fabrication, the PbS solution was purified by a 1:1 mixture of acetone and methanol. This process was repeated three times before the PbS CQDs were re-dispersed in hexane with a concentration of 25 mg/mL.

### Synthesis of ZnO NPs

The ZnO nanoparticles were prepared by a modified method based on a published work [18]. 1.77 g of zinc acetate dihydrate was dissolved in 75 mL of methanol at 65 °C, and 0.89 g of potassium hydroxide was dissolved in 39 mL of methanol. The potassium hydroxide solution was slowly added to the zinc acetate solution during vigorous stirring, and the mixture was kept stirring at 65 °C. The mixture solution became cloudy and then became transparent again within 10 min. After 1.5 to 2 h, the solution became turbid, and it indicates the ZnO NPs start to precipitate. The reaction stopped after 1 h since the solution became turbid. The ZnO NPs were washed twice with methanol and dissolved in a 2:1 mixture of chloroform and methanol with a concentration of 100 mg/mL.

### DBR fabrication

TiO_2_ layers and SiO_2_ layers of the DBR film were deposited alternately on a glass substrate at 500 °C using sputtering. TiO_2_ layers were sputtered by direct current (DC) sputtering of a Ti metal target while introducing oxygen to the argon-ion flow. SiO_2_ layers were sputtered by radio frequency (RF) sputtering of a SiO_2_ target. Each optical layer thickness was controlled by adjusting deposition time while the sputtering power was fixed. The 10 nm of Ag thin film was deposited by using the electron beam evaporation system (Thermionics) under vacuum of 10^–6^ Torr at a rate of 0.8 Å/s. Prior to Ag deposition, a 1 nm of Ge layer was deposited to promote adhesion of the Ag layer to the oxide surface of the DBRs. To examine the FP resonance effect, 10 nm of Ag layer on a glass substrate and 200 nm of ITO layer on a glass substrate were prepared as control samples.

### Device fabrication

An 80 nm thick ZnO electron transport layer (ETL) was spin-cast on the bottom electrode layer at 4000 rpm for 40 s. The PbS absorption layer was composed of two parts. The first part was deposited on top of the ZnO layer using a solution-phase ligand exchanged PbS solution. The solution-phase ligand exchange was adapted from a published method [19]. 0.1 M of PbI_2_, 0.02 M of PbBr_2_ and 0.06 M of NH_4_Ac were dissolved in dimethylformamide (DMF). 10 mL of PbI_2_/PbBr_2_/NH_4_Ac solution was added to 10 mL of oleic-acid-capped PbS solution (10 mg/mL in hexane). The mixture was shaken vigorously for around 1 min, and then the PbS CQDs were transferred to the DMF matrix. The upper hexane solution was removed and the DMF solution containing PbS CQDs was washed three times with hexane. After washing, the ligand exchanged PbS CQDs were precipitated using toluene antisolvent and dried under vacuum for 20 min. The PbS CQDs were redispersed in butylamine, and the deposition was performed by a single-step spin coating. The layer thickness was adjusted by making a different concentration of the PbS CQD solution as well as controlling the speed of the spin-coating speed. For the 150 nm thickness of the PbS layer, a solution with a concentration of 120 mg/mL was used, and the spin-coating was performed at 2500 rpm for 30 s. After depositing the solution-phase ligand exchanged PbS layer, two PbS-EDT layers were deposited using a layer-by-layer spin-coating [20] as a second part of the PbS absorption layer. For each spin-coating cycle, 50 µL of PbS solution was spin-cast on the prepared substrate at 2500 rpm for 30 s. For an EDT ligand exchange process, 100 µL of 0.02 vol% EDT/acetonitrile solution was spin-cast for 30 s. Residual EDT was washed away using three-time acetonitrile rinsing. From each spin-coating cycle, a 25 nm thick PbS layer was obtained. The top MAM structure was deposited through the electron beam evaporation under vacuum of 10^–6^ Torr at a rate of 1 Å/s for MoO_3_ and 0.8 Å/s for Ag. MoO_3_, Ag and MoO_3_ were deposited in series to form the conductive and transparent DMD contact. The active area of the top electrode of the solar cells was 0.175 cm^2^.

### Characterization

J–V characteristics were measured using simulated solar light (Oriel Sol 3A, Newport) and an electrochemical workstation (CHI660, CHI Instrument). 1-sun AM 1.5G illumination was calibrated to a reference Si solar cell (PVM 95). The incident photo-to-current conversion efficiency (IPCE) was measured through quantum efficiency measurement kit (Oriel QEPVSI-b, Newport) in air using the light of a 300 W xenon lamp. The transmittance and the reflectance of the films were measured using an UV–Vis spectrophotometer (Lambda 35, PerkinElmer) equipped with an integrating sphere. The conductivity of the thin films was examined using a hall-effect measurement system (HMS-5000, Ecopia) at room temperature. The optical response of the devices was calculated by using software based on the finite-difference time-domain (FDTD, Lumerical) method. Three-dimensional models were used to simulate suggested device structures. Periodic boundary conditions were applied along the x- and y-direction. The plane-wave illuminates from the top of the structure and propagates along the z-direction where perfectly matched layers (PMLs) were applied. The PbS absorption spectra were calculated using FDTD simulations to predict the incident photon to current conversion efficiency of the devices. The incident angle dependent results for non-polarized light were calculated by incoherently summing results from s-polarized and p-polarized calculations. The optical constants and thicknesses of each layer were estimated by combining physical thickness (DektakXT surface profiler, Bruker) and optical thickness (F40 thin film analyzer, Filmetrics).

## Results and discussion

In the design of the device in Fig. 1a, the top transparent electrode is MoO_3_/Ag/MoO_3_ (MAM) multilayer. MoO_3_ is chosen as the dielectric component of the DMD film, since MoO_3_ can be deposited at low temperature to avoid collateral damage of PbS layer. In addition, MoO_3_ film has been studied as an interlayer between a PbS active layer and an anode metal. The high work function of MoO_3_ pins the Fermi level of the metal contact and turns the metal-PbS interface from a Schottky junction to an ohmic junction [21]. Therefore, MoO_3_ in MAM improves the hole extraction efficiency. In the design of the MAM multilayer, it is important to optimize the thicknesses of the dielectric (MoO_3_) and metal (Ag) layers. They control not only the optical transparency and electric conductivity of the transparent electrode but also the FP resonance wavelength [22]. If the MoO_3_ layer is too thin or thick, the efficiency of hole extraction or the fill factor of the solar cell is reduced. After several experimental tests, the thickness of the inner and outer MoO_3_ was fixed at 10 nm and 20 nm, respectively. The thickness of Ag layer of the MAM is also crucial. A thinner Ag layer exhibits better transparency of incident light. However, the Ag thin layer tends to form isolated nuclei at the early stage of the deposition because metal-to-metal interaction is much greater than metal-to-oxide substrate interaction [23, 24]. This discontinuous Ag layer not only reduces electrical conductivity but also impairs optical transparency due to surface plasmon resonance of the island-shaped Ag layer [25]. To quantitatively understand the effect of each layer, the interaction of the light with the MAM structure was calculated using finite-difference time-domain (FDTD). From this simulation, the MAM film was designed to maximize the additional light absorption in visible and near infrared ranges by the FP resonance. In addition to the optical properties of the MAM structure, the sheet resistance of the MAM film was measured to find the thickness of the metal layer that provides the good electric conductivity without considerably attenuating the light intensity in the PbS layer (Additional file 1: Table S1 and Fig. S1). From this simulation, the optimal MAM structure was determined to be 10 nm of inner MoO_3_, 10 nm of Ag, and 20 nm of outer MoO_3_.

**Fig. 2:**
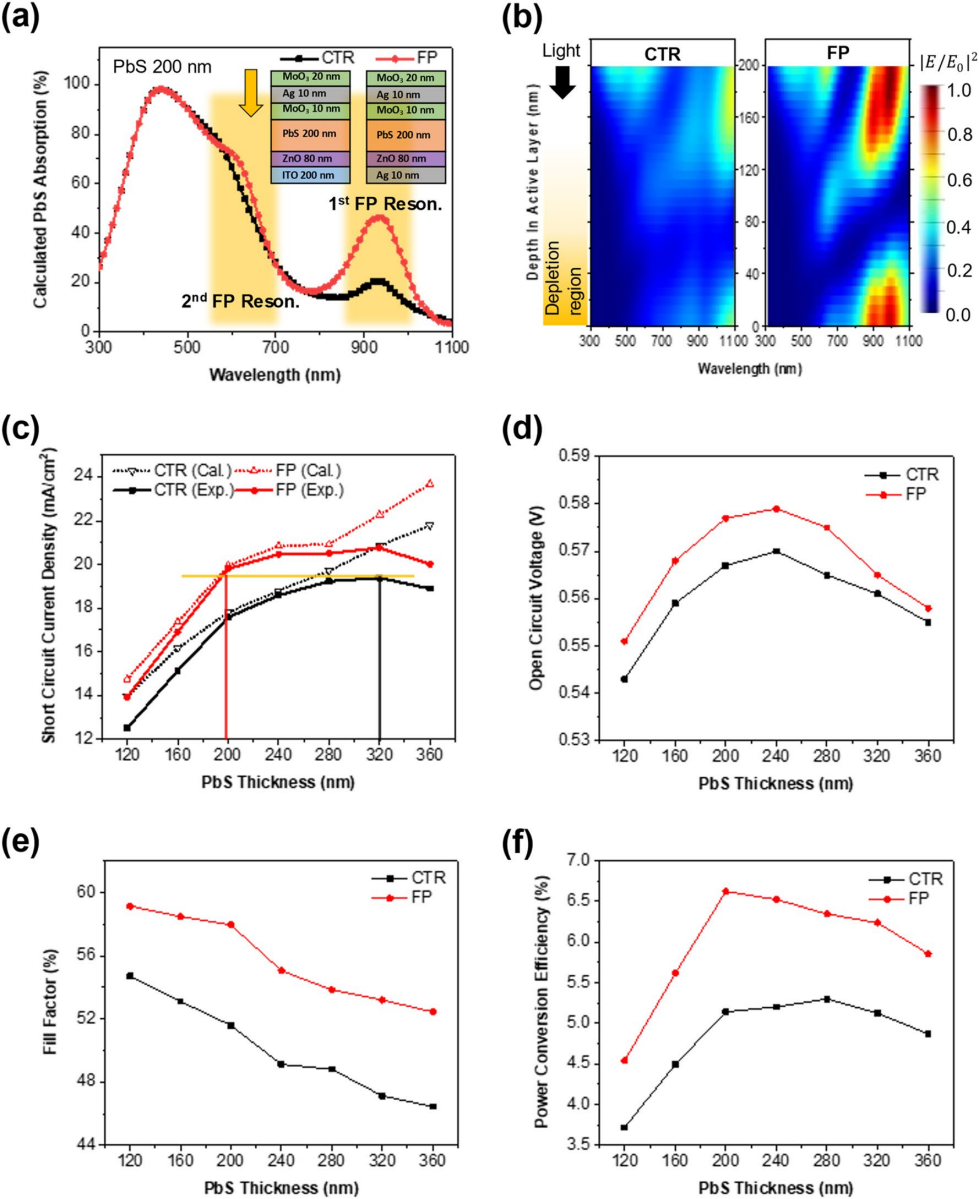
**a** Calculated absorption spectra of 200 nm thick PbS in CTR and FP devices. **b** Normalized electric field intensity in the devices under 1-sun AM 1.5G illumination. **c** J_SC_ of the devices with different PbS active layer thicknesses. The solid lines represent calculated J_SC_ without considering diffusion limits of the charge carriers in the devices, and dotted lines represent experimentally measured J_SC_. Experimental **d** V_OC_, **e** FF, and **f** PCE of the devices

Figure 2a, b show the simulation results on the light absorption of the PbS CQD solar cells using the FP resonance. The bottom electrode of a control device (named as CTR) is 200 nm thick ITO that has a similar sheet resistance as the bottom 10 nm thick Ag layer. In Fig. 2a, the FP resonance clearly increases the light absorption of the CQD layer in the selective wavelength range. With a fixed thickness of the MAM, the ZnO, and the bottom Ag, the resonant wavelength of the FP cavity can be controlled by the thickness of the active CQD layer. As the thickness of the PbS layer increases, the cavity size increases, and the resonant wavelength shifts to a longer wavelength (Additional file 1: Fig. S2). At the given structure including 200 nm thick PbS layer, two FP resonance peaks are observed. The first resonance peak is located at 850–1050 nm which is overlapped with the first absorption peak (at 930 nm) of the PbS CQD (Additional file 1: Fig. S3). The second one is found at 550–750 nm. Enhanced light absorption is observed in both ranges. In Fig. 2b, it is worth noticing that the increase in the electric field by the first FP resonance is very pronounced near the bottom PbS/ZnO interface where the depletion region is formed to facilitate the electron–hole dissociation. Thus, more charge carriers are successfully extracted and collected via the drift at the depletion region of the FP resonance device. This results in an increase in the internal quantum efficiency as well as the IPCE.

Figure 2c shows simulation results of short-circuit current density (J_SC_) vs. PbS thickness on the assumption that all photogenerated carriers are converted to electric current (i.e., internal quantum efficiency = 100%) and experimental results. In contrast to the simulation results, the 320 nm thick PbS layer exhibits the maximum J_SC_. The overall light absorption enhancement pushes quasi-Fermi level to a higher energy level due to increase of photogenerated electrons, resulting in increase of the built-in potential and V_OC_. Excessive carriers can fill defects sites and reduce V_OC_ deficit caused by trap states. The dependence of photocurrent on the PbS thickness is different between simulation and experimental results because the internal quantum efficiency is assumed to be 100% in the simulation. However, the carrier trapping—recombination of real devices makes this value less than 100%. As the PbS layer becomes thicker than the charge carrier diffusion length, the internal quantum efficiency decreases more dramatically [20]. This, in turn, decreases fill factor (FF) and open-circuit voltage (V_OC_), as shown in Fig. 2d, e. Also, if the PbS thickness is thinner than the charge carrier diffusion length, the IPCE and FF are still high but the amount of absorbed light is reduced, leading to a decrease in J_SC_ and V_OC_ [26]. Because of a tradeoff between Jsc and Voc as a function of the PbS layer thickness, the highest efficiency is found at the 280 nm thick PbS layer (CTR) and 200 nm (the FP resonance solar cells). A smaller critical thickness of the FP device is due to the localized electric field enhancement which is schematically explained in Fig. 2b. Since the electric field intensity is increased near the top and bottom interfaces of the PbS layer in the FP resonance solar cells, a thinner layer of PbS layer between MAM and the bottom Ag can absorb more light without increasing the recombination probability. Note that there is negligible variation in the photovoltaic performance of the CTR device with respect to the illumination direction in our case. Although the transmittance of ITO in the broad band spectrum is relatively higher than that of MAM, a substantial amount of optical loss is observed in the visible region due to parasitic absorption caused by the sol–gel ZnO layer which is located between ITO and PbS when the light comes from the ITO side. In contrast, MAM illumination shows less optical loss in the visible region while the surface reflectance in NIR region is high. As a result, the amount of light absorbed by PbS itself does not vary significantly depending on the illumination direction (Additional file 1: Fig. S4).

**Fig. 3:**
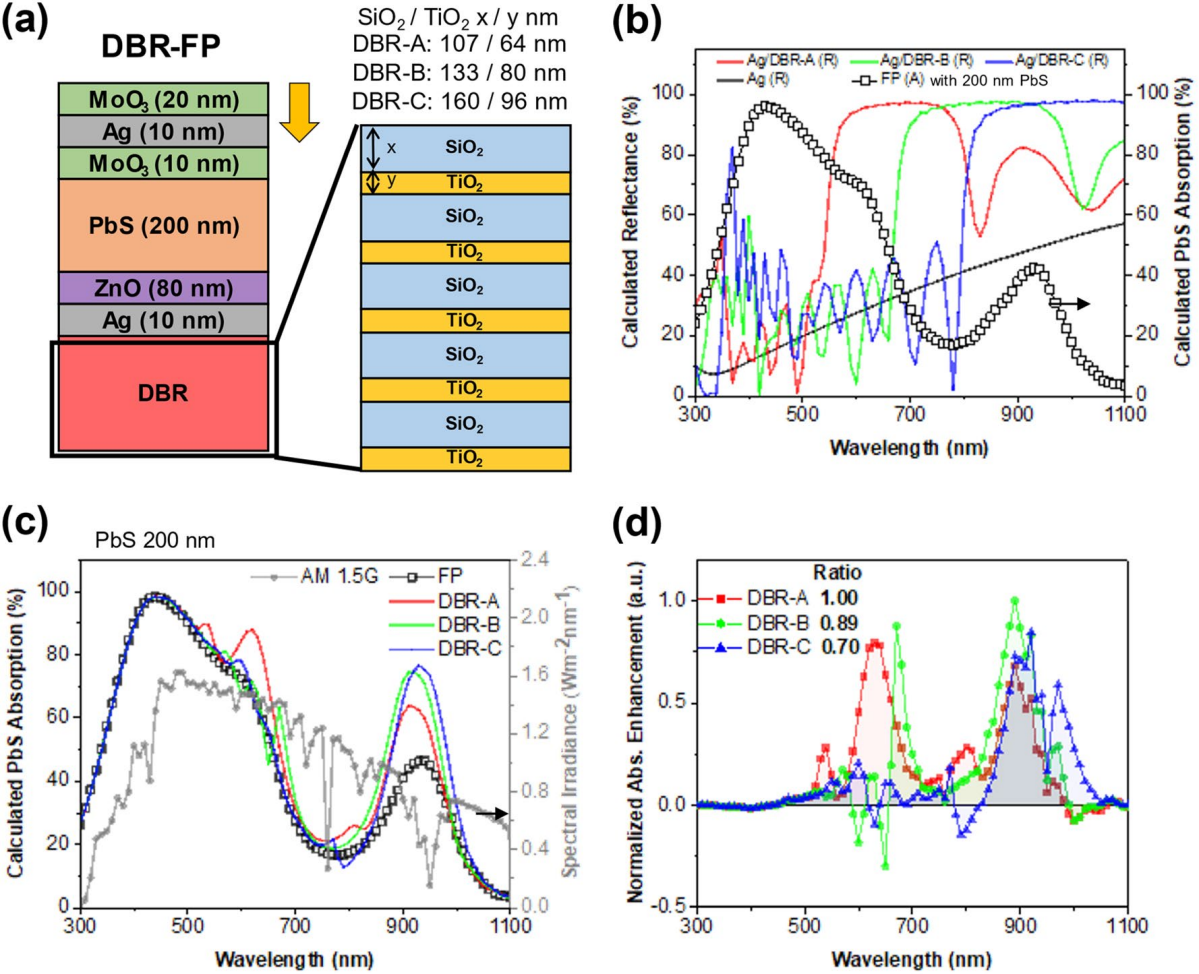
**a** Schematic of DBR-FP device. **b** Calculated reflectance of DBR-A, DBR-B, DBR-C, and absorption spectra of 200 nm thick PbS in the FP device without a DBR. **c** Calculated absorption spectra of 200 nm thick PbS in DBR integrated FP devices, and **d** corresponding normalized absorption enhancement of the devices under AM 1.5G illumination

Though the FP resonance is observed, the device structure of Fig. 2a has a limitation of low reflectance. Since the bottom Ag layer is very thin (10 nm), it only reflects about 30% of incident light in the range of 300–1100 nm. To further promote the FP resonance effect, the highly reflective DBR structure of alternate films is added under the bottom Ag electrode (Fig. 3a). TiO_2_ (n ~ 2.5) and SiO_2_ (n ~ 1.5) are chosen because of the large difference in the refractive index. The center resonant Bragg wavelength λ_R_ and the spectral width ∆λ_R_ of the high reflectance zone are calculated using Eqs. (1) and (2), where n is the refractive index and d is the film thickness [27, 28].

1 $${\lambda }_{R}=2\left({n}_{\text{SiO}_{2}}\times {d}_{\text{SiO}_{2}}+{n}_{\text{TiO}_{2}}\times {d}_{\text{TiO}_{2}}\right)$$

2 $$\frac{\Delta {\lambda }_{R}}{{\lambda }_{R}}=\frac{4}{\pi }\times {\mathrm{sin}}^{-1}\left(\frac{{n}_{\text{TiO}_{2}}-{n}_{\text{SiO}_{2}}}{{n}_{\text{TiO}_{2}}+{n}_{\text{SiO}_{2}}}\right)$$

When TiO_2_ is directly under the Ag bottom layer, the structure is abbreviated as DBR-TS, and the opposite structure in which SiO_2_ is under the Ag layer is abbreviated as DBR-ST. As shown in the electric field distribution in Additional file 1: Fig. S5, the incident light is reflected most at the top of the bottom Ag layer in the DBR-ST structure. In a case of the DBR-TS structure, however, a strong Tamm plasmon is excited between the bottom Ag layer and the top TiO_2_ layer of the DBR. The strong absorption under the bottom Ag layer leads to absorption loss in the active layer. For this reason, DBR-ST is chosen for enhancing the absorption of the active PbS layer. The effect of the number of the DBR pairs was also simulated. Although more TiO_2_–SiO_2_ pairs increase the overall absorption of the DBR added devices more, an increment of the light absorption becomes marginal from the 5 TiO_2_–SiO_2_ pairs (Additional file 1: Fig. S6). Hence, all DBR-FP solar cells have 5 TiO_2_–SiO_2_ pairs with a top Ag layer.

To explore the selective light reflectance of DBRs and its effect on the photocurrent, FDTD simulations are performed for three types of DBRs (Additional file 1: Fig. S7). The thickness of the dielectric layer is changed to control the resonant wavelength of the DBR. The center resonant wavelength of the DBR-A is 640 nm which overlaps with the second FP resonance wavelength. The center resonant wavelength window of the DBR-B is 800 nm which overlaps with the low absorption valley of the PbS CQDs. The center resonant wavelength of the DBR-C is 960 nm, which is overlapped with the first FP resonance wavelength and the PbS excitation peak (Fig. 3b). Figure 3c shows the calculated absorption. The enhancement of the absorption under AM 1.5G is also normalized in Fig. 3d. The DBR-A exhibits the most enhancement. The DBR-A increases the reflectivity of the bottom Ag layer at the wavelength of the second FP resonance peak. Comparison of Figs. 2a, 3b, and d suggests that the coupling of the DBR resonant wavelength with the FP resonance wavelength is very important to increasing the photocurrent of the DBR-FP solar cells.

**Fig. 4:**
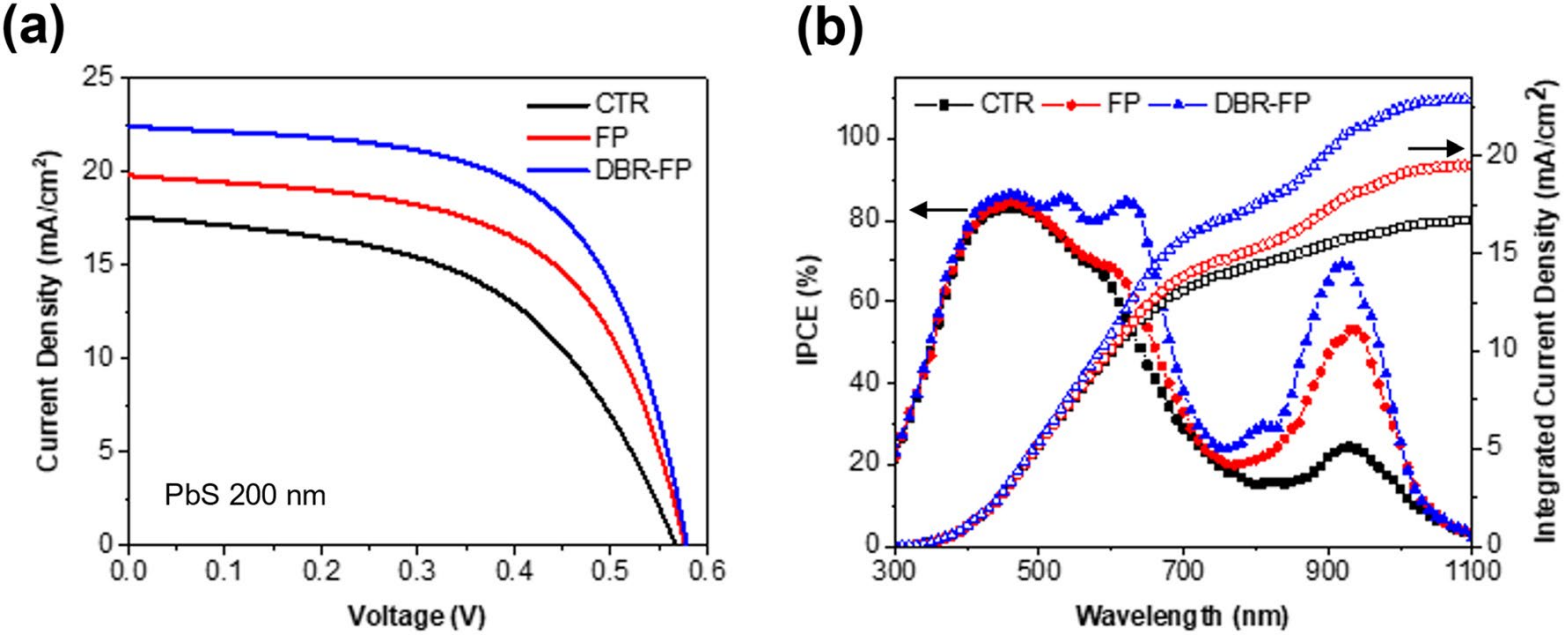
**a** J–V curves and **b** IPCE spectra of CTR, FP, and DBR-FP devices (PbS thickness = 200 nm)

Based on the simulation, CTR, FP, and DBR-FP solar cells are fabricated using the DBR-A design. The thickness of the PbS active layer is fixed at 200 nm. Their photovoltaic performance is compared in Fig. 4, Additional file 1: Fig. S8, and Table 1. Compared to the CTR device J_sc_ increases by 14% for the FP device and 29% for the DBR-FP device. As the photogenerated electrons increase due to the FP and DBR effect, V_OC_ and FF also increase by pushing the quasi-Fermi level to a higher energy level close to the conduction band edge. Figure 4b shows the IPCE spectrum of FB and FP-DBR. The experimentally measured IPCE spectrum of FP and DBR-FP solar cells are in good agreement with the results of the FDTD calculation (Additional file 1: Fig. S9). As predicted, FP resonances of FP-DBR are strongly coupled with the band edge absorption and the supra bandgap absorption of PbS CQDs at 960 nm and 640 nm to increase the IPCE. Such consistent results indicate that the carrier recombination is negligible in FP and DBR-FP devices and an increase in the light absorption is key in boosting their photovoltaic performance. Such an increase in J_SC_ is responsible for 29% and 54% increase of PCE in the FP and DBR-FP device. It is worth noting that the increase of a fill factor can be attributed to the relatively low shunt resistance. The power loss through shunt resistance in the CTR device is relatively high. As light absorption increases in FP and DBR-FP devices, the fraction of the total current flow through the shunt resistance decreases, resulting in a relatively significant increase of fill factor.

**Table 1:** Represented photovoltaic properties of CTR, FP, and DBR-FP devices (PbS thickness = 200 nm)

Device	J_SC_ (mA/cm^2^)	V_OC_ (V)	FF (%)	R_Shunt_ (kΩ cm^2^)	R_Series_ (Ω cm^2^)	PCE (%)
CTR	17.6	0.567	51.6	0.27	7.2	5.14
FP	19.8	0.577	58.3	0.30	4.7	6.67
DBR-FP	22.4	0.579	61.2	0.32	4.2	7.94

**Fig. 5:**
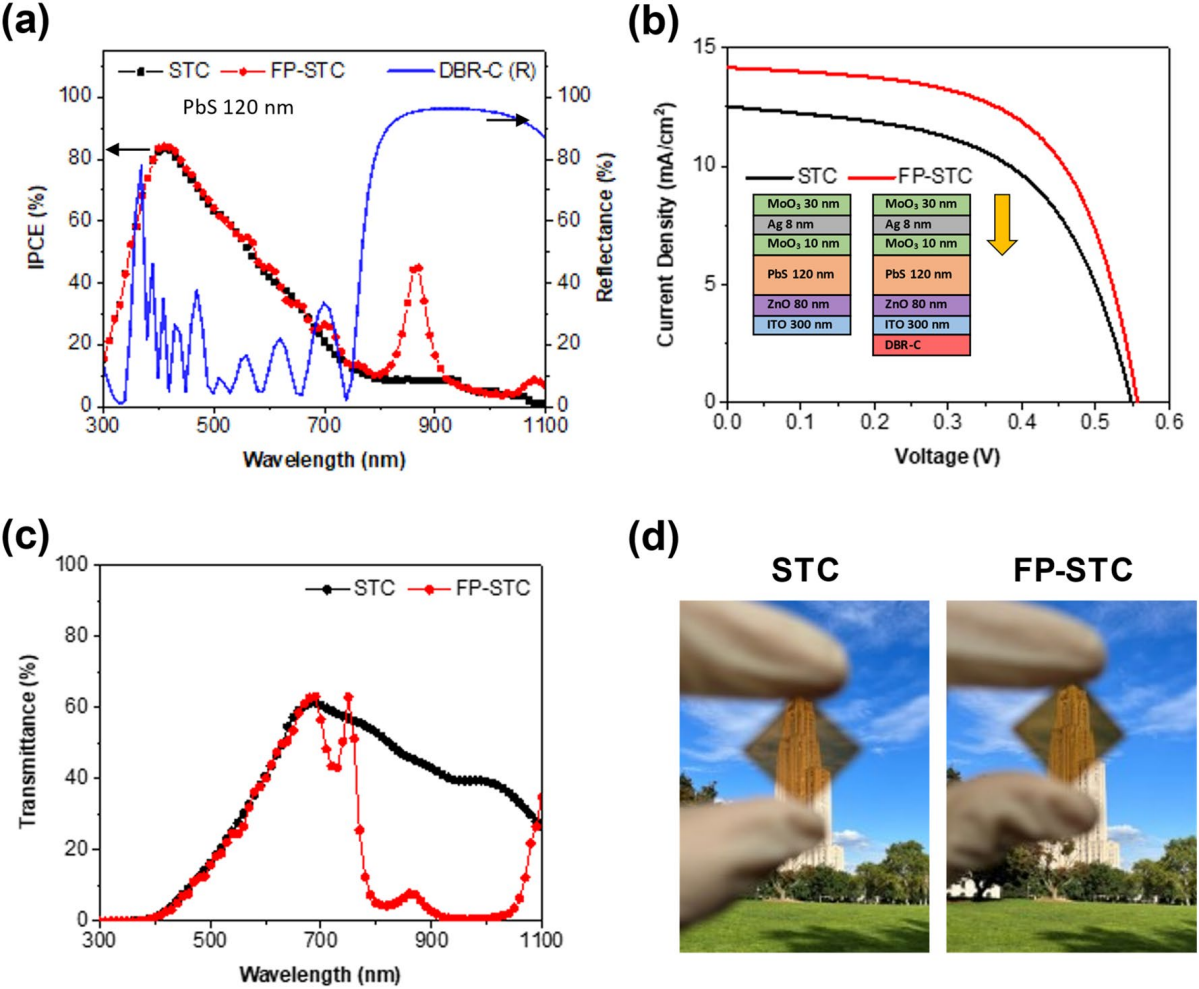
**a** IPCE spectra, **b** J–V curves, and **c** transmittance spectra of STC and FP-STC devices. **d** Photograph of corresponding devices. The thickness of each layer was re-adjusted to maximize visible light transmission. The top MAM layer consists of 30 nm outer MoO_3_, 8 nm Ag interlayer, and 10 nm inner MoO_3_. The PbS thickness is 120 nm. DBR-C, which mainly reflects NIR, consists of 5 pairs of 160 nm SiO_2_ and 96 nm TiO_2_

Since the DBR assisted FP resonance effect enables CQD solar cells to selectively harness near infrared (NIR) light, this structure can be applied to developing a semi-transparent solar cell. To test the feasibility of a new class of semi-transparent solar cells, we fabricated a very thin PbS based device with a combination of MAM top electrode and the DBR-C. This solar cell exhibits a high reflectance for the NIR, but a high transmittance for the visible light. A control sample is a standard PbS CQD solar cell (named as STC) consisting of the MAM (inner MoO_3_/Ag/outer MoO_3_ = 10/8/30 nm) top electrode and a 300 nm thick ITO bottom electrode. The top MAM layer is optimized to maximize the average transmittance (AVT) as shown in Additional file 1: Fig. S10. In DBR assisted FP resonance integrated semi-transparent PbS CQD solar cell (FP-STC), DBR-C in Fig. 3 was added under ITO For both devices, the PbS thickness is fixed at 120 nm. In Fig. 5a, b and Table 2, the photovoltaic performances of STC and FP-STC devices are presented. The STC device shows a very low IPCE in the NIR region because the PbS layer is too thin. When the FP resonance is formed by adding the NIR reflecting DBR, IPCE at the resonant wavelength increases by 440%. This causes a significant increase of J_SC_ and a marginal increase of V_OC_ and FF. Figure 5c shows the transmittance spectra of STC and FP-STC devices. AVT in the range of 380–740 nm remains at almost the same level while the transmittance in the NIR region is significantly reduced. As a result, PCE of FP-STC is larger than that of STC by 24% while both samples have a similar AVT (~ 30%). Figure 5d confirms that the scenery through these devices is similar despite a considerable difference in PCE.

**Table 2:** Represented photovoltaic properties of STC and FP-STC devices (PbS thickness = 120 nm)

Device	J_SC_ (mA/cm^2^)	V_OC_ (V)	FF (%)	PCE (%)	AVT (%)
STC	12.51	0.549	56.1	3.85	31.2
FP-STC	14.15	0.556	60.7	4.77	30.1

It is worth noting that the device structure proposed in this work is sensitive to the incident light angle because the change of the effective optical path length depending on the incident angle affects resonance spectra of the FP resonance and DBRs [29–32]. As shown in Additional file 1: Fig. S11, the PbS absorption in STC and FP-STC decreases as the incident light angle increases. Although the current generation is reduced at a higher angle, the FP-STC device still shows a higher current density compared to the STC device. Note that the FP resonance wavelength at 860 nm in the FP-STC shifts toward a short wavelength as the incident light angle increases. This is because the optical path length through the FP resonance structure changes [29]. An increase in the incident angle leads to a longer effective optical path length, resulting in the blue shift of resonance wavelength. Similarly, the reflectance band of the DBR shifts toward a shorter wavelength region as the incident angle increases [30–32]. As a result, the average visible transmittance (380–740 nm) of the FP-STC device which has an FP resonance between the top Ag layer and the bottom DBR decreases more quickly compared to the STC which does not have a FP resonance. Additionally, in the case of the FP-STC structure, a more pronounced color variation with respect to the incident angle is observed compared to the STC structure (Additional file 1: Fig. S12). While our semi-transparent PbS CQD solar cells utilized a DBR that reflects NIR light to minimize color changes in the visible range, it is worth noting that by adjusting the resonance wavelength of the FP and DBRs, there is a potential to induce a diverse range of color variations in the device based on the incident light angle.

## Conclusions

We have demonstrated the combined effect of the distributed Bragg reflector (DBR) and the Fabry–Perot (FP) resonance for a PbS CQD solar cell. In a FP device, two reflecting electrodes generated a Fabry–Perot resonance in the PbS active layer, and more light was absorbed at two different resonant wavelength regions. Because of this enhanced light absorption, more photocurrent was generated even in a thinner PbS active layer, which led to a higher power conversion efficiency (PCE) by avoiding the decrease in fill factor caused by increase in thickness. In addition, the optical enhancements were further boosted by adding DBRs. Owing to the tunable reflection spectrum of the DBRs, the FP resonance can be strongly coupled to a high reflectance region of the DBRs. This coupling is exploited to compensate for the insufficient short-wavelength reflectance of the thin silver bottom electrode of the CQD solar cells. Consequently, the DBR-FP CQD solar cells show a significant PCE boost by increasing both J_SC_ and FF. By tuning the FP resonance wavelength and the device structure, semi-transparent PbS CQD solar cells with enhanced NIR light absorption can be fabricated without sacrificing optical transparency. The optical engineering demonstrated in this study can be further applied to different optoelectronic devices which need to increase the photon-to-electron conversion efficiency in a certain wavelength range without increasing the thickness of an active layer.

## Supplementary Information

**Additional file 1.** Additional table and figures.

## Data Availability

All data generated or analysed during this study are included in this published article and its additional information files.
