# Supplementary material for: Optical engineering of PbS colloidal quantum dot solar cells via Fabry–Perot resonance and distributed Bragg reflectors
Source: Nano Converg. 2023 Jul 4;10:31. doi: 10.1186/s40580-023-00379-1 (PMC10319680; doi:10.1186/s40580-023-00379-1)
Supplement: Supplementary file 1 — Additional file 1. Additional table and figures. [file 40580_2023_379_MOESM1_ESM.docx]

**Additional Information**

**Optical Engineering of PbS Colloidal Quantum Dot Solar Cells via Fabry-Perot Resonance and Distributed Bragg Reflectors**

Sumin Bae, Matthew Duff, Jun Young Hong and Jung-Kun Lee*

Department of Mechanical Engineering & Material Science, University of Pittsburgh, Pittsburgh PA 15261, USA

* Corresponding author

Email: jul37@pitt.edu

**Table S1.** Sheet resistance of electrodes used for the device fabrication.

| **Electrode** | **Thickness (nm)** | **Sheet Resistance (Ω/□)** |
| --- | --- | --- |
| **ITO** | 200 | 9.23 $\pm$ 0.35 |
| **ITO** | 300 | 5.49 $\pm$ 0.17 |
| **Ag** | 12 | 7.11 $\pm$ 0.47 |
| **Ag** | 10 | 9.45 $\pm$ 0.82 |
| **Ag** | 8 | 22.57 $\pm$ 1.24 |
| **MoO_3_/Ag/MoO_3_** | 10/12/20 | 6.72 $\pm$ 0.43 |
| **MoO_3_/Ag/MoO_3_** | 10/10/20 | 8.09 $\pm$ 0.51 |
| **MoO_3_/Ag/MoO_3_** | 10/8/20 | 17.49 $\pm$ 0.86 |


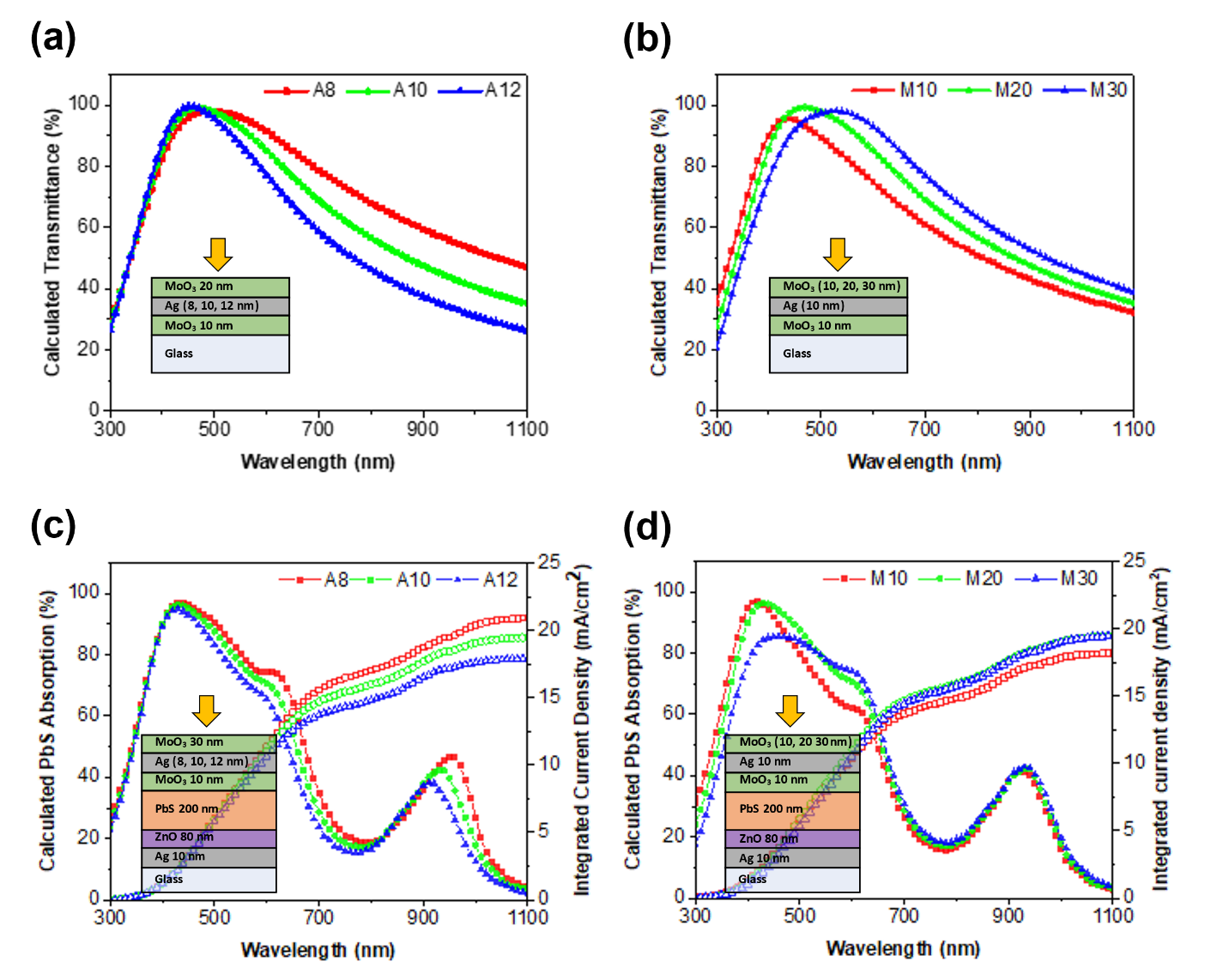


**Fig. S1.** Calculated transmittance spectra of MoO_3_/Ag/MoO_3_ (MAM) layers (a) with different Ag thickness (the Ag thickness is 8 nm, 10 nm, and 12 nm for A8, A10, and A12, respectively) and (b) with different top MoO_3_ thickness (the top MoO_3_ thickness is 10 nm, 20 nm, 30 nm, and 40 nm for M10, M20, M30, and M40, respectively). Calculated absorption spectra of 200 nm thick PbS film and integrated current density of the device using (c) MAM of the different Ag thickness, and (d) MAM of the different top MoO_3_ thickness.


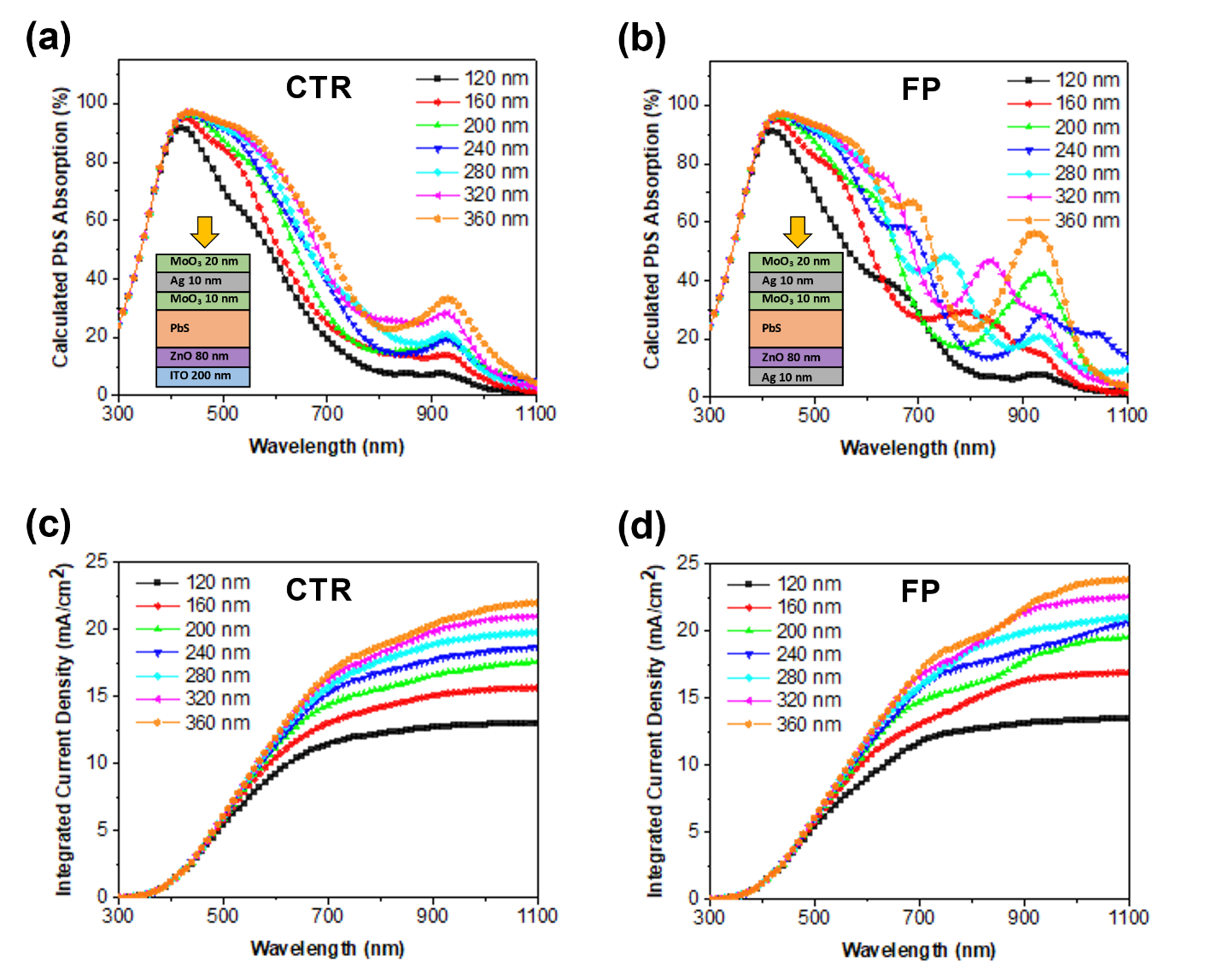


**Fig. S2.** Calculated absorption spectra of PbS active layer in (a) CTR devices, and in (b) FP devices as a function of PbS layer thickness (the top transparent electrode: the bottom - 10 nm thick MoO_3_ layer, the middle - 10 nm thick Ag layer, the top layer - 20 nm thick MoO_3_ layer, the bottom electrode: the 10 nm thick Ag layer). Corresponding integrated current densities of (c) CTR devices, and (d) FP devices.

**Fig. S3.** Absorption spectrum of PbS CQDs in hexane solution as a function of the precursor injection temperature.


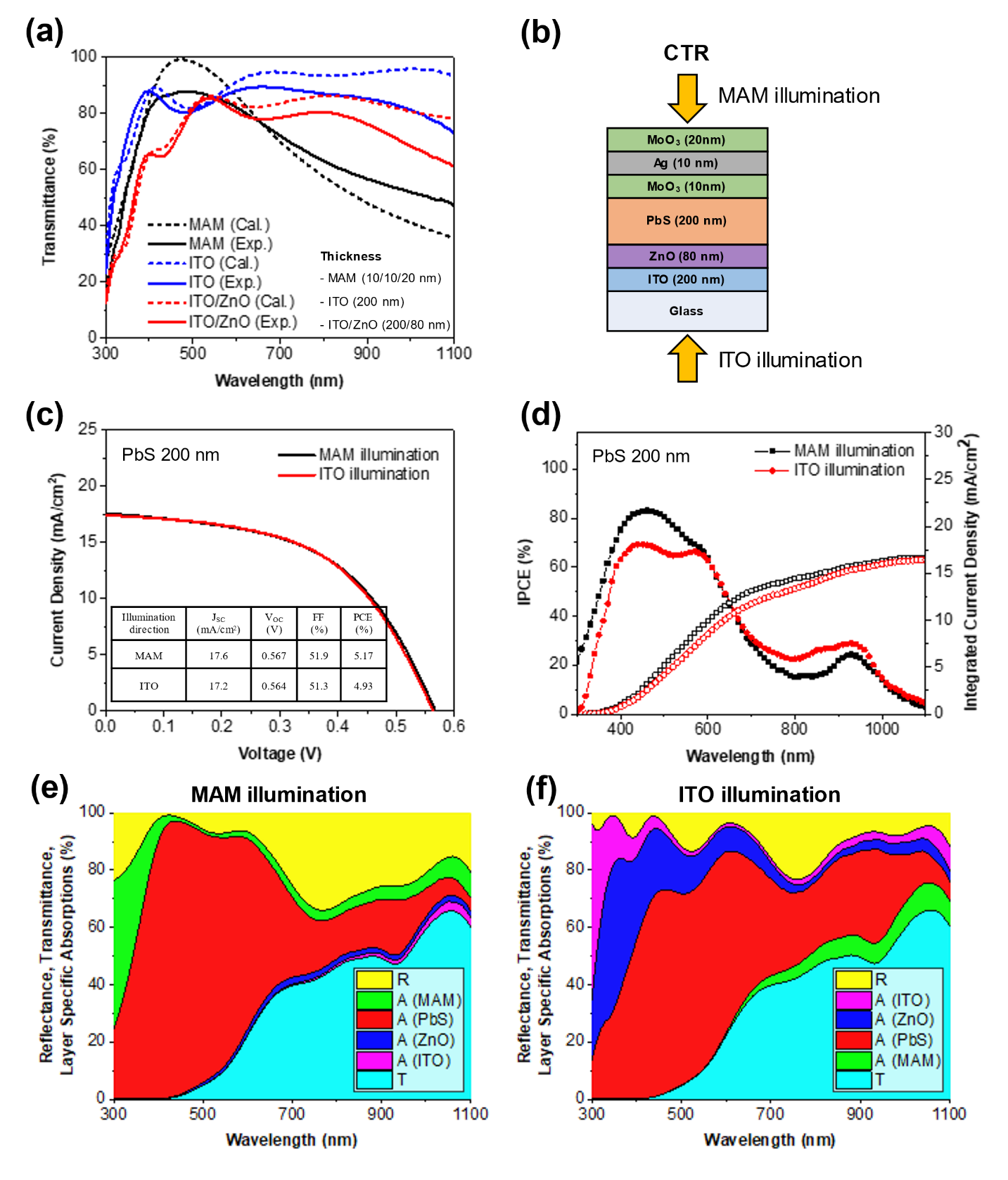


**Fig. S4.** (a) Calculated and experimental transmittance spectra of MAM (10/10/20 nm), ITO (200 nm), and ITO/ZnO (200/80 nm) on glass substrates. (b) Schematic of illumination direction on the CTR device (200 nm thick PbS), and corresponding (c) J-V curves, and (d) IPCE. Calculated reflectance, transmittance, and layer specific absorptions of (e) MAM illumination, and (f) ITO illumination of the CTR devices.


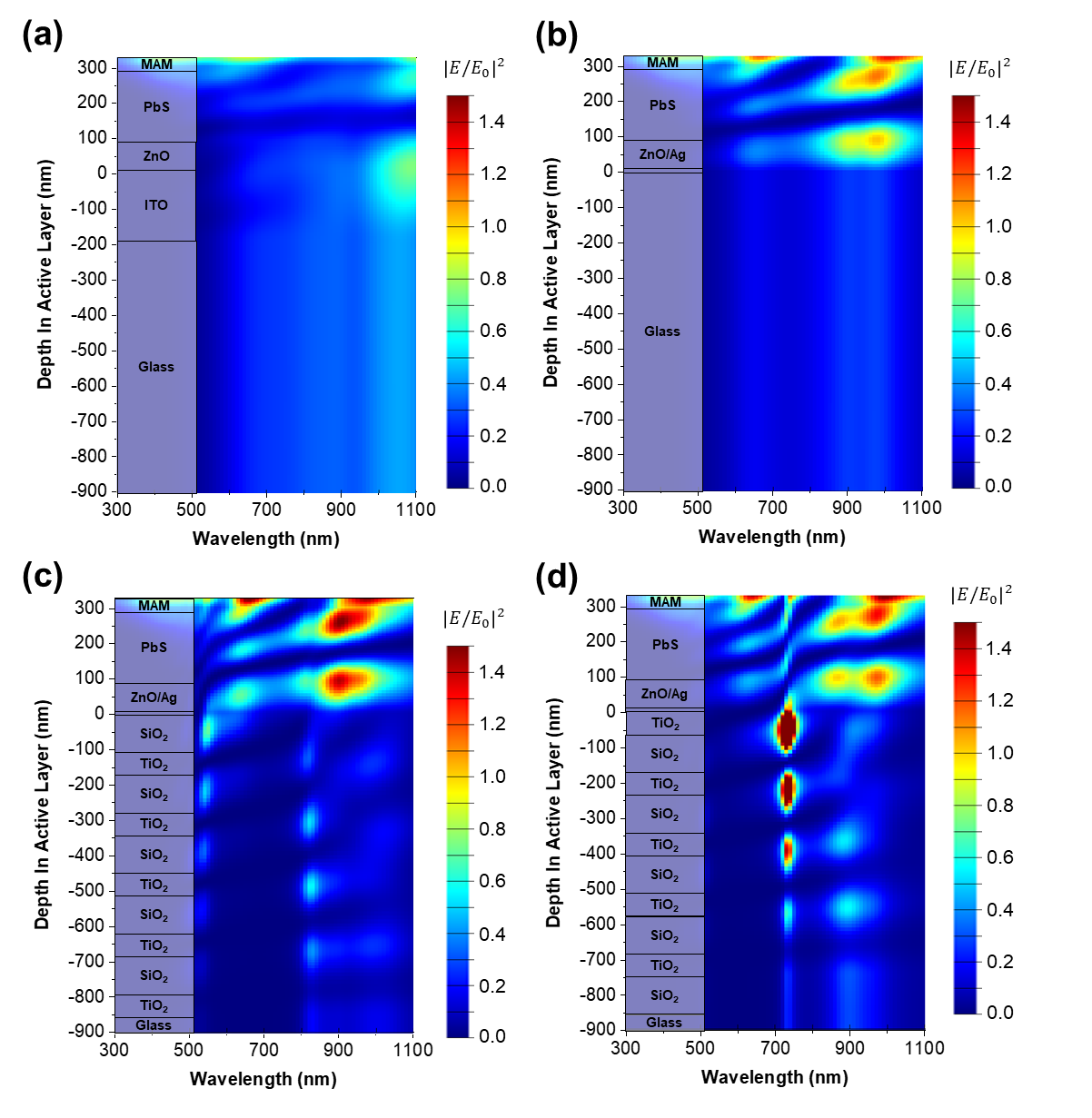


**Fig. S5.** Relative electric field intensity from the FDTD simulation: (a) CTR device, (b) FP device, (c) FP device with DBR-ST, and (d) FP device with DBR-TS under AM 1.5G illumination.


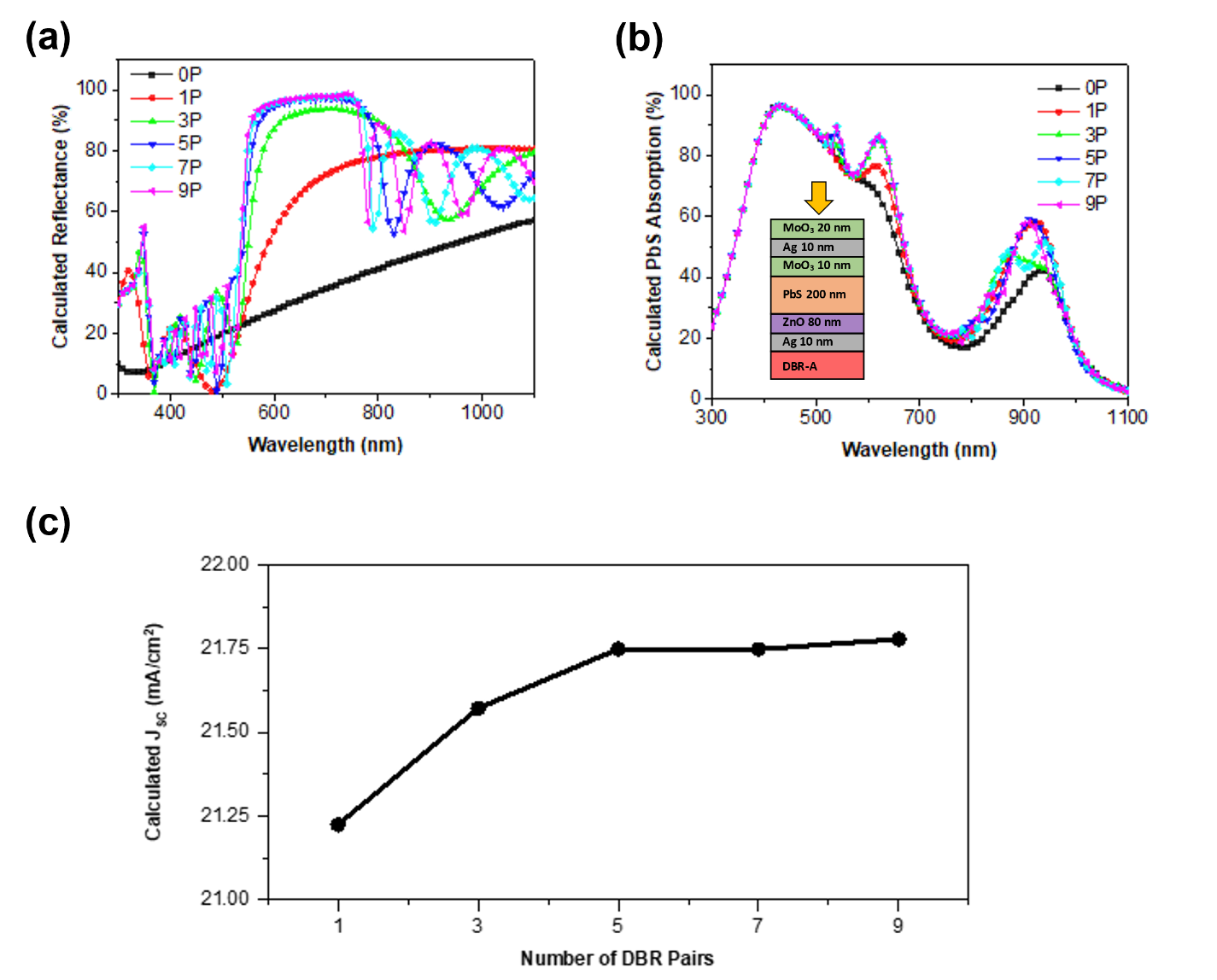


**Fig. S6.** (a) Calculated reflectance of the Ag-coated DBR with a different number of the SiO_2_/TiO_2_ pairs. (b) Calculated absorption spectra of PbS layer (200 nm) on the Ag-coated DBR with a different number of the SiO_2_/TiO_2_ pairs and (c) calculated short-circuit current densities from the absorption spectra.

**Fig. S7.** Reflectance of DBR with a different layer thickness; (a) DBR-1 (SiO_2_/TiO_2_ = 174 nm/64 nm), DBR-2 (SiO_2_/TiO_2_ = 133 nm/80 nm) and DBR-3 (SiO_2_/TiO_2_ = 160 nm/96 nm).


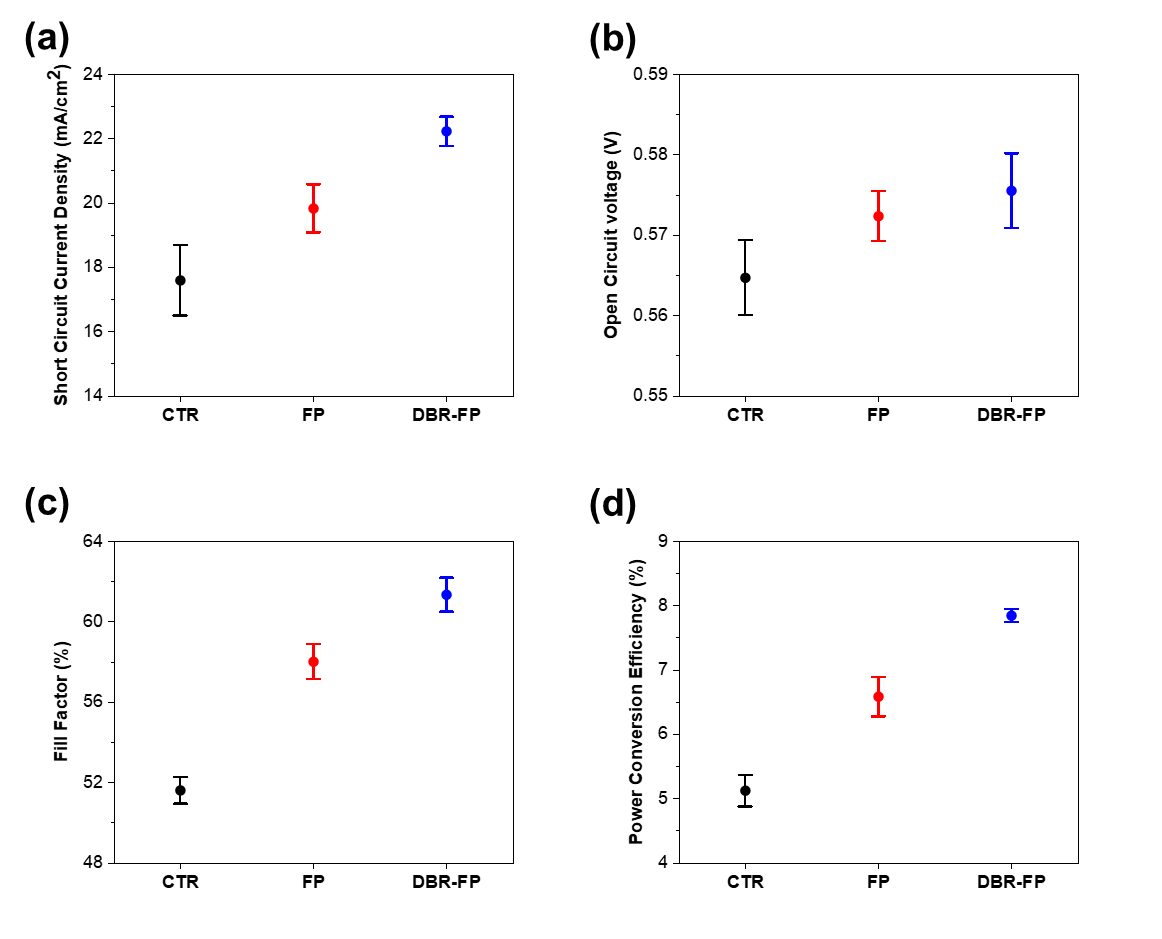


**Fig. S8.** (a) J_SC_, (b) V_OC_, (c) FF, and (d) PCE of CTR, FP, and DBR-FP devices (PbS thickness = 200 nm).

**
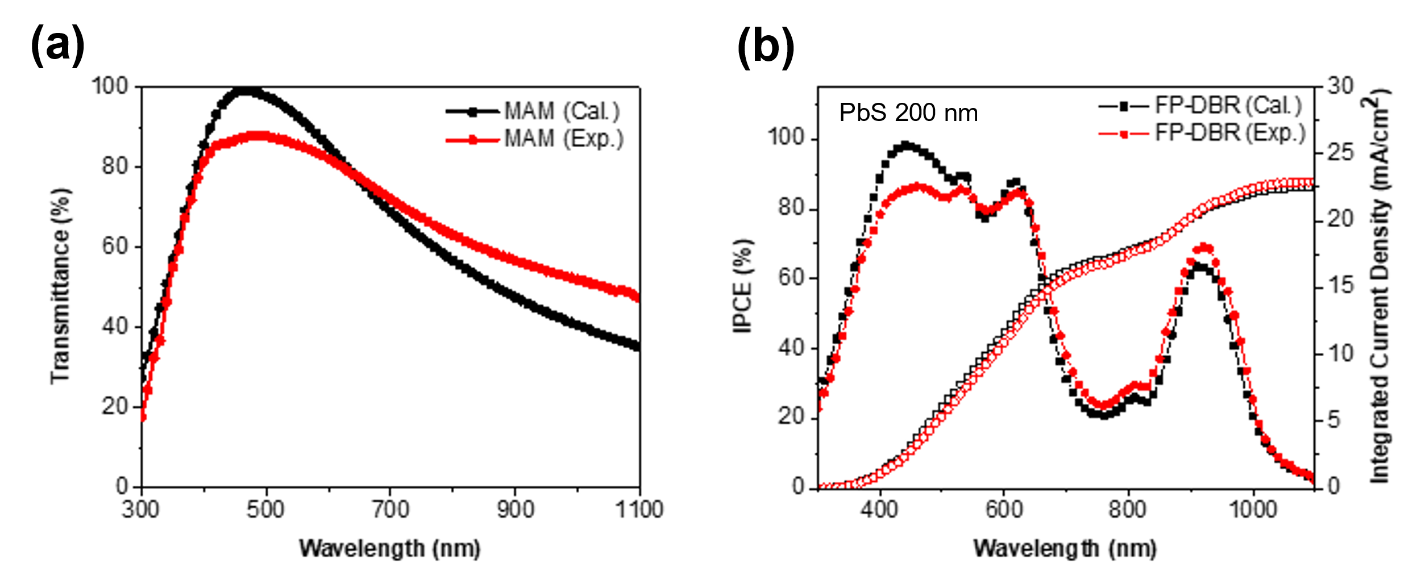
**

**Fig. S9.** (a) Transmittance spectra of the MAM top electrode on a glass substrate, and (b) IPCE spectra of the PbS 200 nm thick FP-DBR device with calculated and experimental results. Note that IPCE the difference is caused by the transmittance difference of the MAM top transparent electrode.


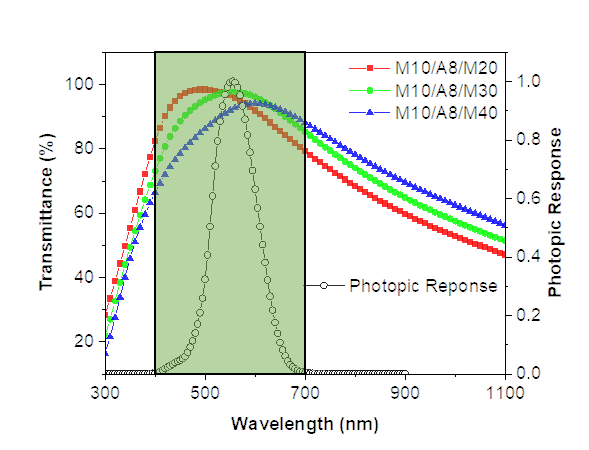


**Fig. S10.** Calculated transmittance spectra of MoO_3_/Ag/MoO_3_ (MAM) layers depending on the outer MoO_3_ thickness with a fixed Ag thickness. The Ag thickness is 8 nm for A8. The outer MoO_3_ thicknesses are 20 nm,30 nm, and 40 nm for M20, M30, and M40, respectively. The integration of the transmittance spectrum against the photonic response of the human eye is maximized at M10/A8/M30.


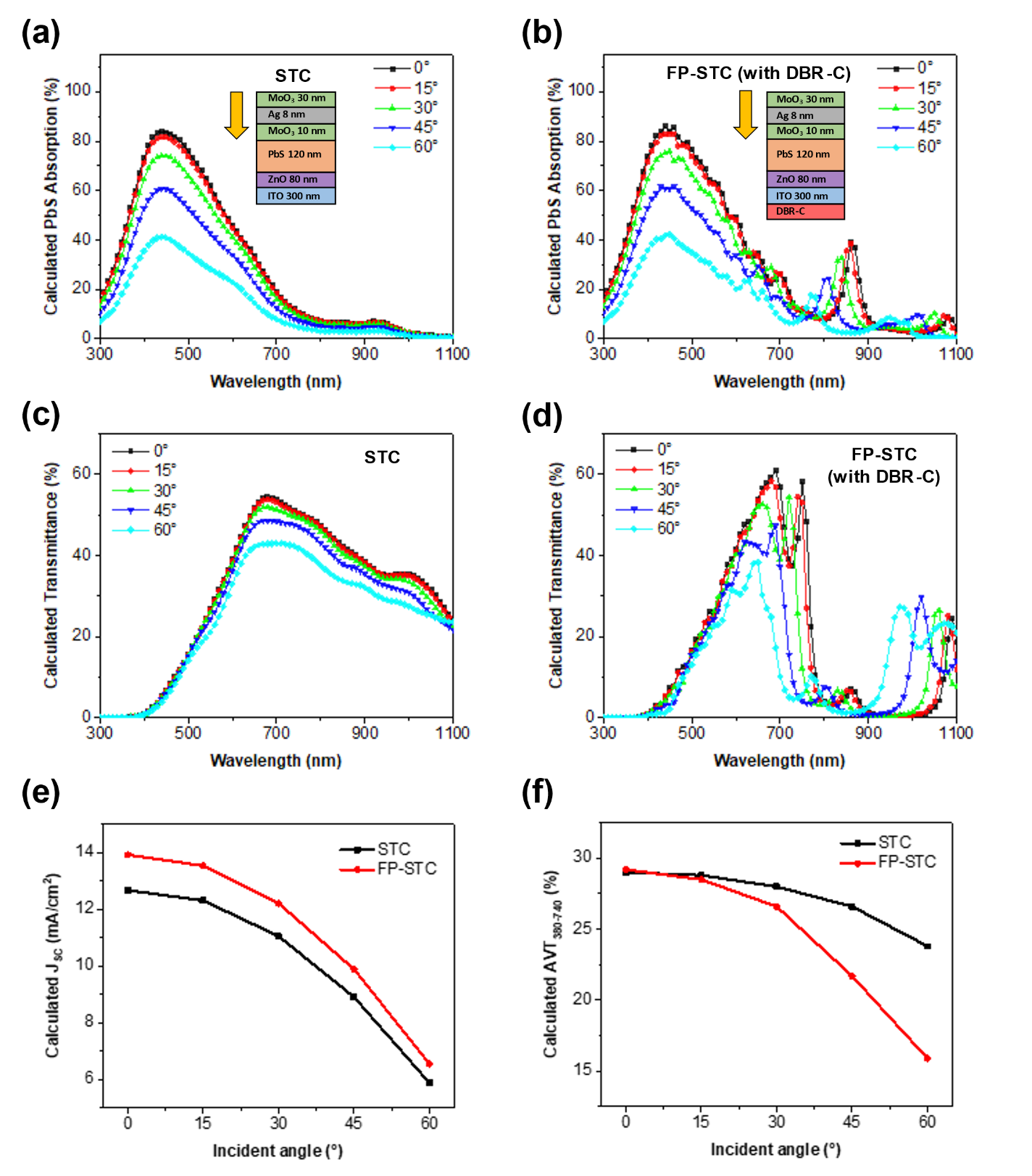


**Fig. S11.** Calculated PbS absorption of (a) STC device and (b) FP-STC device depending on the incident light angle. Corresponding calculated transmittance of (c) STC device, and (d) FP-STC device. (e) Calculated short circuit current density (J_SC_), and (f) average visible transmittance (AVT, 380 – 740 nm) of STC and FP-STC devices. The FP-STC includes DBR-C which consists of 5 pairs of 160 nm thick SiO_2_ and 96 nm thick TiO_2_. The optical responses presented in this figure are calculated results based on non-polarized light.


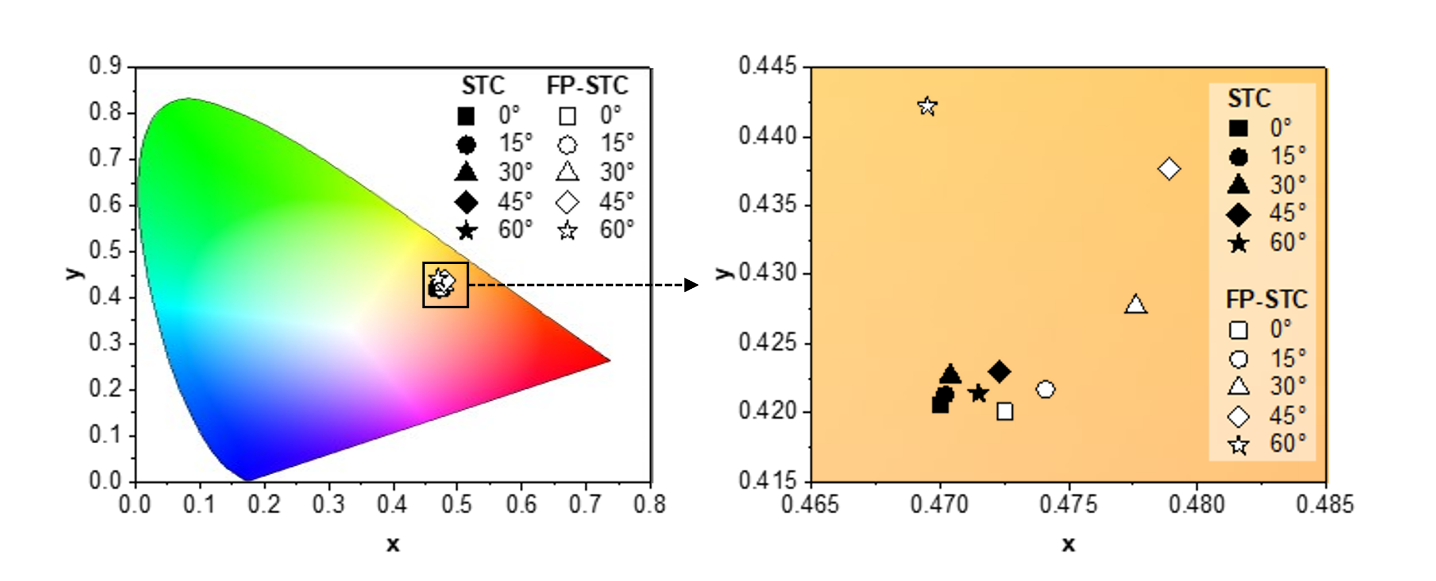


**Fig. S12.** Color coordinates of transmitted light through STC and FP-STC devices on the CIE 1931 chromaticity diagram depending on the incident light angle.
